# Defoliation of Soybean Expressing Cry1Ac by Lepidopteran Pests

**DOI:** 10.3390/insects9030093

**Published:** 2018-08-01

**Authors:** María G. Murúa, Martín A. Vera, María I. Herrero, Sofia V. Fogliata, Andrew Michel

**Affiliations:** 1 Instituto de Tecnología Agroindustrial del Noroeste Argentino (ITANOA), Estación Experimental Agroindustrial Obispo Colombres (EEAOC), Consejo Nacional de Investigaciones Científicas y Técnicas (CONICET), Av. William Cross 3150, Las Talitas T4104AUD, Tucumán, Argentina; maria_inesherrero@hotmail.com (M.I.H.); sofiavfogliata@gmail.com (S.V.F.); 2 EEAOC, Las Talitas 4001, Tucumán, Argentina; alejandrovera_afs@yahoo.com.ar; 3 Department of Entomology, Ohio Agricultural Research and Development Center, The Ohio State University, Wooster OH 44691, USA; michel.70@osu.edu

**Keywords:** *Helicoverpa* genus, *Spodoptera* complex, nontarget and target pests of Bt soybean, seed treatment, leaf damage percentage

## Abstract

Lepidoptera, stink bugs, and weevils are important pests in soybean. For lepidopteran control, insecticides and seed treatments are used. As an alternative, Bt soybean was developed to control primary pests of Lepidoptera such as *Rachiplusia nu* (Guenée) (Noctuidae), *Chrysodeixis* *includens* (Walker) (Noctuidae), *Anticarsia gemmatalis* Hübner (Erebidae), *Helicoverpa gelotopoeon* (Dyar) (Noctuidae), and *Crocidosema aporema* (Walsingham) (Tortricidae). However, the use of transgenic plants, and the resulting reduction of insecticide against target pests, may allow other pest species to become more prevalent in agricultural environments. Soybean expressing Cry1Ac against different lepidopteran nontarget and target insect pests was evaluated, and its performance was compared with non-Bt soybean with seed treatment. The treatments were Bt soybean, non-Bt soybean with seed treatment (Fortenza^®^ diamide insecticide, Syngenta, Buenos Aires, Argentina), and non-Bt soybean without seed treatment. Larvae of *H. gelotopoeon*, *Spodoptera albula* (Walker) (Noctuidae), *Spodoptera cosmiodes* (Walker) (Noctuidae), *Spodoptera eridania* (Stoll) (Noctuidae), and *Spodoptera frugiperda* (J. E. Smith) (Lep.: Noctuidae) were used. The plants of each treatment were infested with larvae of each species, and the percentage of leaf damage produced by each species was recorded. The results showed that Bt soybean provided control of *H. gelotopoeon* and had a suppressive effect on *S. frugiperda* and *S. albula*. However, *S. eridania* and *S. cosmiodes* were not susceptible to the Cry1Ac protein in MON 87701 × MON 89788 soybean when evaluated by greenhouse infestation. Considering the performance of each species using non-Bt soybean without seed treatment, *S. eridania* would represent a potential risk in soybean crops.

## 1. Introduction

Soybean (*Glycine max* (L.) Merrill) is an oilseed plant of the family Fabaceae that is widely planted in various countries worldwide [1]. Argentina is the third-major soybean producer in the world, covering an area of 19.2 million hectares [2,3]. Lepidoptera, stink bugs (Hem.: Pentatomidae), and weevils (Col.: Curculionidae) are important pests that inflict the major yield loss of this crop [1,4,5,6,7,8]. For Lepidoptera in soybean, insecticides and seed treatments are used, but *Bacillus thuringiensis* (Berliner 1915) soybean (Bt soybean) is a new alternative method to control several species [5,6,7,9,10]. Bt soybean is a stacked varietal line developed by Monsanto that combines the transformation events MON 87701 (expressing Cry1Ac protein) and MON 89788 (glyphosate tolerance) [11]. At present, Bt soybean provides control for the primary Lepidopteran pests of soybean such as *Rachiplusia nu* (Guenée) (Lep.: Noctuidae), *Chrysodeixis includens* (Walker) (Lep.: Noctuidae), *Anticarsia gemmatalis* Hübner (Lep.: Erebidae), *Helicoverpa gelotopoeon* (Dyar) (Lep.: Noctuidae), *Crocidosema aporema* (Walsingham) (Lep.: Tortricidae), *Colias lesbia* (F.) (Lep.: Pieridae), *Spilosoma virginica* (F.) (Lep.: Arctiidae), *Chloridea virescens* (F.) (Lep.: Noctuidae), and *Achyra bifidalis* (F.) (Lep.: Crambidae). This technology can also suppress populations of *Spodoptera frugiperda* (J. E. Smith) (Lep.: Noctuidae), *Elasmopalpus lignosellus* (Zeller) (Lep.: Noctuidae), and *Helicoverpa zea* (Boddie) (Lep.: Noctuidae) [11]. However, the use of Bt plants and the resulting reduction of insecticide use against target pests may allow other pest species to become more relevant in agricultural environments [9]. Field trials conducted in northern China showed that mirid bugs (Het.: Miridae) have progressively increased population sizes and acquired pest status in cotton and multiple other crops, in association with a regional increase in Bt cotton adoption. Bt cotton has become a source of mirid bugs, and their population increases are related to drops in insecticide use in this crop [12]. On the other hand, the emergence of *Striacosta albicosta* (Smith) (Lep.: Noctuidae), as a potential pest of corn in South Dakota (USA) may be related to the widespread planting of Cry1Ab Bt corn hybrids. Continuous planting of Cry1Ab Bt corn hybrids over large areas favors this species by effectively eliminating competition from *Ostrinia nubilalis* (Hübner) (Lep.: Crambidae) [13]. Therefore, research on the interaction of nontarget pest species with this Bt technology is of great theoretical and practical importance.

In recent years, the occurrence of caterpillars from the *Spodoptera* genus (Lep. Noctuidae) has increased and caused damage to soybean crops in Brazil [14,15,16,17,18] and Argentina [5,6,7,19]. Within this complex, *S. cosmiodes* (Walker), *S. eridania* (Stoll), and *S. frugiperda* are prominent in causing damage [7,16,20]. It is important to highlight that the damage produced by *S. frugiperda* in soybean crops is common in farms with grasses and pasture weeds before soybean sowing. Some of these grasses and various pasture weeds [*Conyza bonariensis* (L.) Cronquist (Asteraceae), *Brassica campestris* (L.) Metzg. (Brassicaceae), and *Sphaeralcea bonariensis* (Cav.) Griseb (Malvaceae)] play an important role as reservoirs for insect pests, which move from one to another species of grasses or crops [21,22]. The presence of *S. albula* (Walker) in soybean crops is occasional and they were also detected in farms with grasses and pasture weeds. 

The bioecological characteristics of the *Spodoptera* complex (polyphagia, great voracity in feeding, high reproductive capacity, migration behavior, host races) [23,24,25,26,27,28,29,30,31] added to some precedents of insecticide resistance and the natural tolerance of *Spodoptera* spp. to the Cry1A protein [32,33], making it necessary to evaluate their behavior with Bt soybean. Previous studies that evaluated the susceptibility and tolerance of different pests suggested that the most susceptible species to the Cry1Ac protein are *A. gemmatalis*, *R. nu*, and *C. includens*, and the most tolerant species are *S. frugiperda* and *S. cosmioides* [34,35]. While *S. frugiperda* has developed resistance to Cry1F proteins [36,37,38], *S. eridania* and *S. albula* are tolerant to several chemical insecticides [39,40,41,42,43,44] and to the *B. thuringiensis* Cry1Ac gene [45,46].

Given the agronomic challenges of soybean production combined with the emerging *Spodoptera* pest complex, evaluations of additional control strategies are needed. Although various aspects of *Spodoptera* spp. performance have been studied using different crops or artificial diet through field and laboratory studies (Table 1), many of the field studies used natural infestations where the specific identity of each species was not considered. This is due to the fact that the first larval instars of this genus, in general, present similar characters which make it impossible to identify [46,47,48,49]. This has led to much speculation about the damage of this complex in the field. The knowledge about the defoliation produced by each species will help to determine the real individual defoliation in soybean plants.

*Helicoverpa gelotopoeon* is another major pest of soybean. Larvae of this species cause damage in the vegetative and reproductive plant growth stages. In Tucumán and other provinces of Argentina, this species causes severe damage to soybean and chickpea (*Cicer arietinum* L. (Fabaceae)) crops and can be difficult to control with insecticides [50,51,52,53]. Some other species of *Helicoverpa*, such as *H. armigera* Hübner and *H. zea*, have also developed resistance to insecticides and Cry proteins [54,55,56,57,58,59]. 

Previous studies were conducted under field conditions considering the *Helicoverpa* genus in general [7,8,19]. This has led to much speculation, given the coexistence of *H. gelotopoeon* and *H. armigera* in soybean crops in northwestern Argentina [52]. In this genus, species can only be separated by morphological characters of the adults [50]. Thus, the study of *H. gelotopoeon* performance is important to know the real potential of defoliation in soybean crops.

The great potential for defoliation of soybean plants [16,20] and damage to flowers and pods [14] by *Spodoptera* spp. and *H. gelotopoeon* [51,52] requires the adoption of control tactics to prevent yield loss. Control is achieved with insecticides, often indirectly as result of sprays for *A. gemmatalis*, *C. includens*, and *R. nu* [5,6,7,8,60].

The rapid adoption of Bt soybean in Argentina [2] and other countries in South America [61] has increased the need to know the behavior of nontarget and target pests of Bt soybean. The objective of this study was to evaluate the performance of soybean expressing Cry1Ac against different lepidopteran nontarget and target insect pests and to compare its performance with that of a non-Bt soybean with seed treatment. We addressed this study by evaluating the defoliation produced by different lepidopteran pests recorded at eight days after infestation.

## 2. Materials and Methods

### 2.1. Larval Collection

Larvae of *H. gelotopoeon*, *S. albula*, *S. cosmiodes*, *S. eridania*, and *S. frugiperda* were collected from January to March 2014 in commercial soybean fields in La Cocha (Dpto La Cocha) and Overo Pozo (Dpto Cruz Alta) counties in Tucumán province (Argentina). A minimum of 250 larvae (from 3rd to 6th instars) of each species were collected using a vertical beat sheet [62]. Then, each larva was placed in a glass tube (12 cm H and 1.5 cm D) with leaves of soybean and transported to our laboratories at the Estación Experimental Agroindustrial Obispo Colombres (EEAOC). The collected larvae were placed in growth chambers under controlled conditions (27 ± 2 °C, 70 ± 5% RH, 14:10 h L:D) until adult emergence. Late larval instars and adults were examined using morphological characters both to confirm the identity of species [47,49,63,64] and to establish pure cultures for each species in the laboratory. Voucher specimens for each species were deposited in the collection of Sección Zoología Agrícola, (EEAOC) Tucumán, Argentina.

### 2.2. Insect Rearing

Each species colony was maintained in the same chamber under identically controlled conditions at 27 ± 2 °C, 70 ± 5% RH, 14:10 h L:D. Colonies were reared according to the methodology described by Murúa et al. [65] and Herrero et al. [53]. Twenty-five pairs per cage and per species (25 females and 25 males) (*N* = 6) were used. Adults were maintained in cylindrical oviposition cages made out of plastic mesh (20 cm high and 15 cm in diameter) lined with polyethylene bags as an oviposition substrate. For aeration, both ends of the cage were covered with a nylon cloth. The food was provided via a cotton plug saturated with a mixture of honey and water (1:1 v/v) which was replaced every day. Cages were checked daily for oviposition and adult mortality. Eggs were collected daily with a moistened brush and deposited in Petri dishes lined with moistened filter paper. Once emerged, neonate larvae were placed in 20-cm diameter, 800-mL containers with an artificial larval diet that included bean flour (Grandiet^®^, Buenos Aires, Argentina), wheat germ (Grandiet^®^, Buenos Aires, Argentina), brewer’s yeast (Calsa^®^, Tucumán, Argentina), vitamin C (Anedra^®^, Buenos Aires, Argentina), sorbic acid (Anedra^®^, Buenos Aires, Argentina), vitamin supplement amino acids (Ruminal^®^, Buenos Aires, Argentina), and methylparaben (Todo Droga^®^, Córdoba, Argentina). Diet was replaced every five days. As larvae pupated, pupae were sexed and placed in cup containers with moistened filter paper until adult’s emergence. Adults were used to initiate a new generation. After establishing a colony for each species, individuals from the 2nd generation (F2) were used for the evaluation of soybean Cry1Ac against different pest species in a greenhouse.

### 2.3. Greenhouse Studies

Soybean seeds of maturity group 7 were used (recommended for cultivation in northwestern Argentina). The treatments evaluated were Bt soybean Cry1Ac (T1), non-Bt soybean plus seed treatment (T2) (Fortenza^®^ diamide insecticide, Syngenta, Argentina) according to the dose/rate recommended by the company (36 g a.i./100 Kg/seed), and non-Bt soybean without seed treatment as an untreated control (T3). The three treatments were planted in different pots (15 cm D, 600 mL) using sterilized soil. One seed per pot was planted and each plant was labeled to distinguish both the species and treatment. The plants were maintained under greenhouse conditions under ambient lighting at approximately 33 ± 4 °C, 80 ± 10% RH, 14:10 h L:D.

The experimental design was completely randomized with three replicates per treatment, where each replicate consisted of 20 soybean plants. A total of 60 plants for each pest species (5) per treatment (3) were evaluated, resulting in 900 plants.

Each plant for each treatment was inoculated at the V1 stage [66] (approximately 14 days after planting) with 10 larvae (L1) of a pest species. The evaluations of defoliation produced by each species were recorded at 8 days after inoculation (DAI), according the visual estimation of defoliation scale described by Kogan and Turnipseed [67].

Expression of the Cry1Ac protein in the soybean plants was confirmed using qualitative ELISA Quickstix lateral flow detection strips (Envirologix, Portland, ME, USA).

### 2.4. Data Analysis

To meet parametric assumptions, percentage data on defoliation damage were transformed to arcsine square root prior to analysis [68]; nevertheless, untransformed means (±SE) are shown in the figure. The percentage of defoliation damage produced by each species in the different treatments and the performance of these species using non-Bt soybean without seed treatment were analyzed using a one-way ANOVA, and means were separated using Tukey’s tests (*p* < 0.05) with InfoStat [69].

## 3. Results

**Figure 1:**
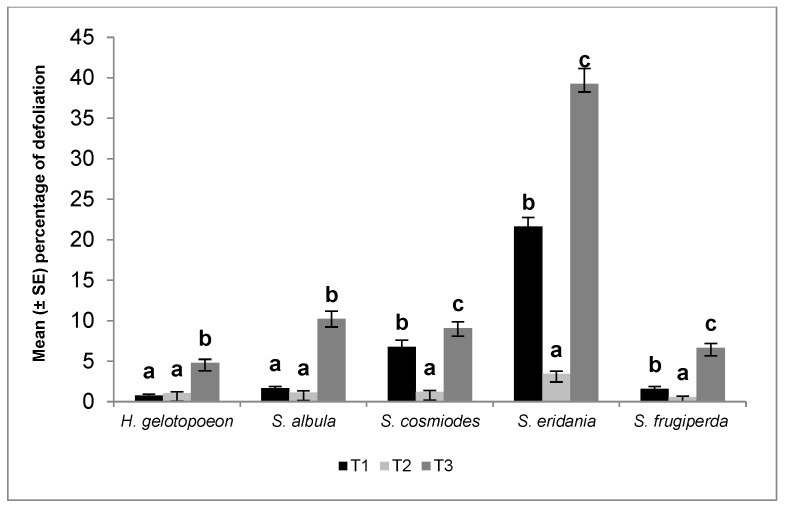
Percentage of defoliation produced by *Helicoverpa gelotopoeon*, *Spodoptera albula*, *Spodoptera cosmiodes*, *Spodoptera eridania*, and *Spodoptera frugiperda* (Lepidoptera: Noctuidae) larvae in different soybean treatments (T1: Bt soybean, T2: non-Bt soybean with seed treatment, T3: non-Bt soybean without seed treatment). Means ± SE within species accompanied by different letters indicate significant differences (Tukey test, *p* < 0.05).

All results are shown in Figure 1. Significant differences were found in the average defoliation damage produced by *H. gelotopoeon* among different treatments (*F* = 64.3; df = 2,177; *p* < 0.0001). Bt soybean (T1) and non-Bt soybean plus Fortenza^®^ (T2) presented similar values, but significant differences were found when they were compared with the control (T3). The average defoliation damage produced by *S. albula* was lower in the Bt soybean and non-Bt soybean plus Fortenza^®^ treatments compared to the control (Figure 1) (*F* = 111.0; df = 2,177; *p* < 0.0001). For *S. cosmiodes*, lower feeding damage was recorded in the non-Bt soybean plus Fortenza^®^ treatment, compared to the Bt soybean and control plants (*F* = 76.0; df = 2,176; *p* < 0.0001). The best control of *S. eridania* was with the non-Bt soybean plus Fortenza^®^ treatment, registering a lower percentage of defoliation compared to Bt soybean and the control plants (*F* = 269.1; df = 2,177; *p* < 0.0001). Finally, significant differences were found in the percentage of defoliation produced by *S. frugiperda* in the different treatments (*F* = 94.7; df = 2,177; *p* < 0.0001). The lowest defoliation was obtained with non-Bt soybean plus Fortenza^®^ (0.57 ± 0.12), whereas the control plants showed the highest defoliation.

A comparison of the potential risk of these species on untreated plants (non-Bt soybean without seed treatment) showed significant differences (*F* = 100.9; df = 4,293; *p* < 0.0001). The highest defoliation damage was produced by *S. eridania* larvae (39.3 ± 1.89), followed by *S. albula* (10.2 ± 0.97), *S. cosmiodes* (9.1 ± 0.79), *S. frugiperda* (6.7 ± 0.54), and *H. gelotopoeon* (4.8 ± 0.43).

## 4. Discussion

This study was conducted to characterize the efficacy of soybean expressing Cry1Ac against *H. gelotopoeon*, *S. albula*, *S. cosmiodes*, *S. eridania*, and *S. frugiperda* and compare its performance with that of a non-Bt soybean with seed treatment by assessing defoliation (Figure 1). On the other hand, the performance analysis made among the species using non-Bt soybean without seed treatment showed which of these species would represent a potential risk for soybean crops. For both analyses, the defoliation and potential risk were calculated considering the identity for each species in greenhouse conditions (Table 1).

Our results showed that Bt soybean provided variable control of some pests evaluated. This new technology had the best control of *H. gelotopoeon* and a suppressive effect on *S. frugiperda* and *S. albula*.

Non-Bt soybean with seed treatment provided good control of the five species. Nevertheless, this control will be only for the early plant growth stage, when plants have more insecticide active ingredient in their system. *S. cosmiodes*, *S. eridania*, *S. frugiperda*, and *S. albula* feeding resulted in the lowest percentage of defoliation compared with the other treatments.

In the case of *S. albula*, the defoliation recorded in T1 was similar to that recorded in T2 and the differences were not significant.

These results are consistent with field studies evaluating Bt soybean in Brazil and Argentina. A study of diversity, composition, and population dynamics of arthropods in non-Bt soybean and Bt soybean showed that *A. gemmatalis*, *C. includens*, *C. virescens*, and *S. frugiperda* were significantly controlled by Bt soybean. However, other species of the *Spodoptera* complex were not controlled by this technology [70]. Other studies reported that *S. cosmiodes* was the most abundant species attacking Bt soybean [71] and that development and reproduction of this species were not affected by the Cry1Ac protein [20]. A field study showed that the lepidopteran pests recorded during the soybean cycle were *R. nu*, *A. gemmatalis*, *H. gelotopoeon*, *S. frugiperda*, and *S. cosmioides* [72] and the population abundance of the last two species was similar in Bt and non-Bt soybean. In northwestern Argentina, a trial conducted to evaluate Bt soybean against different insect pest and natural predators found that this new technology provided good control of target pests such as *A. gemmatalis*, *C. includens*, and *R. nu*. The levels of leaf damage observed with Bt soybean were lowest and significantly different to those obtained with non-Bt soybean [4]. On the other hand, bioassays and greenhouse studies found that *S. cosmiodes*, *S. eridania*, and *S. frugiperda* exhibited low to no susceptibility to MON 87701 × MON 89788 soybean containing the protein Cry1Ac, and these species showed higher tolerance to the Cry1Ac protein than other Lepidoptera species, such as *C. includens*, *C. virescens*, and *H. zea* [60]. In the same study, a moderate larval incidence of *S. eridania* and *S. frugiperda* on MON 87701 x MON 89788 soybean and the respective near-isogenic negative check was found. No significant differences in larval incidence and defoliation by *S. eridania* were found with Bt soybean and the near-isogenic negative checks for both maturity groups. Similarly, larval incidence of *S. frugiperda* on Bt soybean did not differ significantly from the respective near-isogenic negative checks for both maturity groups. However, defoliation by *S. frugiperda* on Bt soybean was significantly lower than on the near-isogenic, being the defoliation registered, similar to those reported in this study in Bt soybean. The defoliation recorded for *S. eridania* was different to those reported in greenhouse studies in Brazil [60], but in both studies, defoliation was high in non-Bt soybean. These differences could be due to the number of larvae used to evaluate the defoliation per plants.

The non-Bt soybean with seed treatment showed the lowest defoliation of *S. frugiperda*, *S. albula*, *S. cosmiodes*, and *S. eridania.* These results are similar to another study [73], where diamides had good potential to control different lepidopteran pests such as *S. eridania*, *S. cosmiodes*, *S. albula*, *A. gemmatalis*, and *S. frugiperda*. 

Considering the performance of each species using non-Bt soybean without seed treatment, *H. gelotopoeon* showed the lowest defoliation. This species is one of the most important pests in soybean crops, but its larvae prefer the reproductive plant growth stages [50,74], which explains the low defoliation recorded in this study. As mentioned, *Spodoptera* spp. are polyphagous, but show preferences to different host plants, such as the preference of *S. frugiperda* to corn (*Zea mays* L. (Poaceae)) over soybean and other crops [75]. *Spodoptera cosmiodes* and *S. albula* presented similar levels of defoliation in our test, although *S. cosmiodes* is the most frequent *Spodoptera* spp. affecting soybean crops during the vegetative and reproductive stages [5,6,14,16,20,50]. Another study suggested that *S. cosmioides* may be more adapted to chemical compounds of soybean and cotton (*Gossypium hirsutum* L. (Malvaceae)), given their faster development and higher survival rates on these hosts [76]. Larvae of *S. albula* feed on a wide variety of host plants and they also exhibit some preference for several weeds (*Boerhavia erecta* (L.) (Nyctaginaceae); *Echinochloa colonum* (L.) Link (Poaceae), from which they can migrate to cultivated plants [27,29]. The highest defoliation damage was produced by *S. eridania*. This species had been cited as infesting a large number of crops in various regions of the Americas [49,74,77]. Additionally, this species has been reported from outbreaks under different conditions [15,18,28]. This species develops on weeds, which generally constitute a primary source of cultivated plant infestations such as those of soybean [15,26,28]. Considering the defoliation recorded in this study, *S. eridania* would represent a potential risk to soybean crops. According to these results, this species may be more adapted to chemical compounds of soybean given their faster development and higher survival rates [76].

## 5. Conclusions

Our results suggest that *H. gelotopoeon*, *S. frugiperda*, and *S. albula* were susceptible to the Cry1Ac protein. However, *S. eridania* and *S. cosmiodes* were not susceptible to the Cry1Ac protein in MON 87701 × MON 89788 soybean when evaluated by greenhouse infestation, supporting the idea that the level of activity of this protein against these species is low. Consequently, other control tactics, such as seed treatment, must be used in combination with MON 87701 × MON 89788 soybean in the field for the efficient management of *Spodoptera* species.

Our results contribute to determining the defoliation and potential risk of these pests in soybean crops. Considering the increasing importance of *S. cosmioides* and *S. eridania* in the region, future studies should be focused toward understanding survival, population dynamics, and infestation of these species during all growth stages of soybean plants, and their biology, including host adaptation. On the other hand, determination of the combined action of Bt with the seed treatment in the defoliation in the field will be important, like other control tactics. Therefore, the use of Bt soybean as a tool for integrated pest management should be planned according to the major insect problems in each area. 

## Figures and Tables

**Table 1:** Summary of previous studies of the *Spodoptera* complex and *Helicoverpa gelotopoeon* (Lep.: Noctuidae), where the performance of each species was evaluated using different crops and/or artificial diet in field and laboratory studies.

Study	Species *	Identity of Species **	Environmental Conditions (Controlled or Field Conditions)	Type of Infestation	Crop or Artificial Diet Used	Evaluations	Impacts
Present study	Sf, Se, Sa, Sc, Hg	individual	controlled (greenhouse)	artificial	soybean (Bt and non-Bt)	Performance and potencial risk	Bt soybean had the best control of Hg and a suppressive effect on Sf and Sa. Se and Sc were not susceptible to the Bt soybean. Se represents a potential risk in soybean crops
[60]	Sc, Se, Sf	individual	controlled	artificial	soybean (Bt and non-Bt) and diet	Susceptibility to Cry1Ac protein	Bt soybean showed poor control of Sc, Se, Sf
[16]	Sf, Se, Sc	individual	controlled	artificial	soybean genotypes	Larva consumption foliage	Sc defoliated nearly twice the leaf area of Sa and Sf
[5]	S. spp.	general	field conditions	natural	soybean	Different chemical alternatives for the management of the complex pest	Diamide + neonicotinoid had the best control
[6]	Sc	individual	field conditions	natural	soybean	Moment of application of different insecticides	Early application of diamide delayed the damage of larvae
[7]	H. spp., S. spp.	general	field conditions	natural	soybean (Bt and non-Bt)	Pest management	Early application of diamide delayed the damage of larvae
[8]	H. spp.	general	field conditions	natural	soybean (Bt and non-Bt)	Strategies for prevention of insect resistance	Low presence of H. spp. in Bt soybean and refuge
[19]	S. spp., H. spp.	general	field conditions	natural	soybean (Bt and non-Bt)	Behavior of Bt soybean on the pests and its predators	Bt soybean provided a good control of S. spp. and H. spp. and did not affect its predators
[18]	Se	individual	controlled	artificial	soybean cultivars (non-Bt)	Development, survival, and reproductive capacity	The development of Se was affected by the cultivar
[73]	Sf, Se, Sa, Sc	individual	controlled	artificial	diet	Susceptibility to chlorantraniliprole and flubendiamide	Chlorantraniliprole showed a higher mortality than flubendiamide for all Lepidoptera species tested
[70]	S. spp., Sf	general	field conditions	natural	soybean (Bt and non-Bt)	Diversity, composition, and population dynamics	Bt soybean reduced the target insect pests and favored populations of natural enemies
[71]	Sf, Sc, Hg	individual	field conditions	natural	soybean (Bt and non-Bt)	Insect abundance	Densities of the species were low in both treatment
[27]	Sa		controlled	artificial	diet	Developmental parameters and host plants	Complete detail of biological parameters of Sa and 55 host plant species of Sa are listed
[26]	Se	individual	controlled	artificial	diet	Biotic potential and reproductive parameters	Complete detail of reproductive and population parameters of Se
[28]	Se	individual	controlled	artificial	diet	Developmental parameters and host plants	Complete detail of biological parameters of Se and 202 host plant species of Se are listed
[29]	Sa		controlled	artificial	diet	Biotic potential, life table parameters and fertility	Complete detail of reproductive and population parameters of Sa
[72]	Sf, Sc, Hg	individual	field conditions	natural	soybean (Bt and non-Bt)	Strategies of refuge management	The management of refuges with selective insecticides and high persistence allowed to reduce the number of applications and to achieve greater survival of predators and adults of target pests
[15]	Se	individual	controlled	artificial	cotton, soybean, and *Ipomoea grandifolia* (L.) (Convolvulaceae)	Biology on different host plant	Soybean was the least suitable for the development of Se, and *I. grandifolia* was shown a suitable alternate host for Se
[20]	Sc	individual	controlled	artificial	corn (Bt and non-Bt), soybean (Bt and non-Bt), and diet	Development and reproduction	Bt and non-Bt corn adversely affect the development of Sc, and Bt soybean did not affect its biology, suggesting that Sc has major potential to become an important pest in Bt soybean crops
[76]	Se, Sc	individual	controlled	artificial	soybean, cotton, corn, *Triticum aestivum* (L.) (Poaceae), *Avena sativa* (L.), and diet	Biology on different host plants	Soybean and cotton were more suitable hosts for the development of Se and Sc

* Species: (only the species involved in the present study are mentioned) S. spp.: *Spodoptera* species; Sf: *S. frugiperda*; Se: *S. eridania*; Sa: *S. albula*; Sc: *S. cosmioides*; H. spp.: *Helicoverpa* species; Hg: *H. gelotopoeon*. ** General: specific identity of each species was not considered in the study; Individual: specific identity of each species was considered in the study.
